# Low‐Power Tunable Micro‐Plasma Device for Efficient and Scalable CO_2_ Valorization

**DOI:** 10.1002/advs.202507687

**Published:** 2025-06-30

**Authors:** Bartu Karakurt, Hongkeng Zhu, Onder Soydal, Guangyu Sun, Jeremy S. Luterbacher, Elison Matioli

**Affiliations:** ^1^ Institute of Electrical and Micro‐engineering Ecole Polytechnique Fédérale de Lausanne (EPFL) Lausanne CH1015 Switzerland; ^2^ Institute of Chemical Sciences and Engineering Ecole Polytechnique Fédérale de Lausanne (EPFL) Lausanne CH1015 Switzerland

**Keywords:** CO_2_ valorization, energy efficiency, micro‐plasma, sustainability

## Abstract

Valorizing CO_2_ into chemical building blocks via efficient electrified processes could significantly decrease carbon emissions. In this work, a device that allows generation of tunable micro‐plasma modes (arc, pulsed, pulsed arc) on a chip for efficient, low‐power and renewable chemical production is presented. This concept is first demonstrated by using nanosecond repetitively pulsed (NRP) micro‐plasma for pure CO_2_ splitting and show that the pulse micro‐plasma maintains a peak energy efficiency of 29% across 0.2%–21% CO_2_ conversion at plasma powers below 1 W‐nearly an order of magnitude lower power than other systems with comparable performance. The kinetic studies feature pulsed arc mode as a novel way to improve CO_2_ conversion, where CO production tripled compared to pulsed mode with negligible energy efficiency losses. In addition, these devices are also capable of performing dry reforming of methane (DRM) with a maximum energy efficiency of 33% and a syngas ratio close 1. The fact that these devices are low‐power, versatile and built as chips enables scalable, sustainable and decentralized CO_2_ conversion.

## Graphical abstract

Tunable in‐chip micro‐plasma generation leads to efficient, low‐power and scalable CO_2_ valorization under atmospheric pressure via vibrational excitation mechanism.

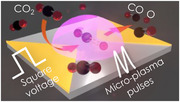

## 1 Introduction

The chemical industry is vital to the functioning of society, producing essential materials that range from detergents to semiconductors.^[^ ^1^ ^]^ However, this sector also imposes a considerable environmental toll, ranking as the second largest oil consumer and third largest emitter of greenhouse gases.^[^ ^2^ ^]^ In this aspect, utilization of CO_2_ as a renewable carbon feedstock draws a lot of attention as it can decrease the sector's dependence on hydrocarbons by allowing generation of a closed‐loop system through carbon capture, utilization and storage (CCUS) technology.^[^ ^3^ ^]^

Recently, there has been a significant effort to find ways to not only utilize CO_2_ as a renewable carbon feedstock but also systematically electrify the carbon dioxide conversion processes since electrification allows utilization of renewable electricity as an energy source.^[^ ^4^, ^5^ ^]^ Therefore, electrification of carbon dioxide valorization techniques has the potential to decrease the carbon emissions related to CO_2_ recycling.^[^ ^6^, ^7^ ^]^

The use of plasmas offers some promising routes for green CO_2_ valorization as it paves the way for energy efficient conversion of CO_2_ into various chemical building blocks (CBBs)^[^ ^8^, ^9^, ^10^, ^11^ ^]^ by utilization of renewable electricity. Nonetheless, efficient atmospheric pressure plasma systems typically require a considerable amount of power. For instance, gliding arc plasma (GAP) is one of the most efficient types of plasmas, where very high CO_2_ splitting efficiencies, ≈45%–50%, have been reported for conversion of carbon dioxide into CO under atmospheric pressures.^[^ ^12^, ^13^ ^]^ Yet, these systems typically operate at high flow rate regimes (on the order of a few Liters/min) to drag the arc along the column,^[^ ^8^ ^]^ thus large power (≈0.1–1 kW) is required for plasma generation. A similar situation is also valid for atmospheric pressure microwave (MW) plasmas, where high energy efficiencies ≈30%, achieved for specific energy input (SEI) ≈5 kJ L^−1^, are obtained for kW plasma powers.^[^ ^14^, ^15^ ^]^ These inherently high plasma powers inevitably cause a substantial portion of wasted energy during the reaction, despite the high energy efficiencies. This makes developing efficient as well as low‐power plasma technologies essential for less energy‐intensive plasma‐mediated CO_2_ valorization.

Here, we introduce a novel plasma device that enables efficient, decentralized and low‐power conversion of carbon dioxide into CBBs by generation of tunable micro‐plasma modes confined to micrometer scales. These devices are driven by a square voltage, and through the precise modulation of their period, duration and frequency, it is possible to achieve a controllable generation of three distinct micro‐plasma phases: *arc*, *pulsed*, and *pulsed arc*. We demonstrate the properties of this device for splitting of pure CO_2_, and show that, through the formation of a sequence of micro‐plasma pulses, it is possible to target the most energy efficient CO_2_ splitting route, namely vibrational excitation mechanism.^[^ ^16^ ^]^ We showcase the scalability of the micro‐plasma technology via simultaneously driving multiple decentralized micro‐plasma devices in a stainless‐steel reactor at room temperature (RT), where an energy efficiency ≈29% is maintained when CO_2_ conversion is increased from 0.2% to 21%, increasing SEI from 0.079 to 9.52 kJ L^−1^. The maximum SEI reported in this work is achieved at plasma powers below 1 W—≈10 times lower than other systems with comparable SEI and efficiency, where typical atmospheric plasmas cannot operate. Finally, we demonstrate the generality of micro‐plasma devices via dry reforming of methane (DRM), that yielded an energy efficiency of 33% along with a CO/H_2_ ≈1, that is desirable for synthesis of oxygenated chemicals.^[^ ^17^ ^]^ The high efficiency, flexibility and scalability of micro‐plasma devices make them an alternative chemical platform for low‐power, sustainable and decentralized green chemical production.

## 2 Micro‐Plasma Device Working Principle and Experimental Setup

**Figure 1:**
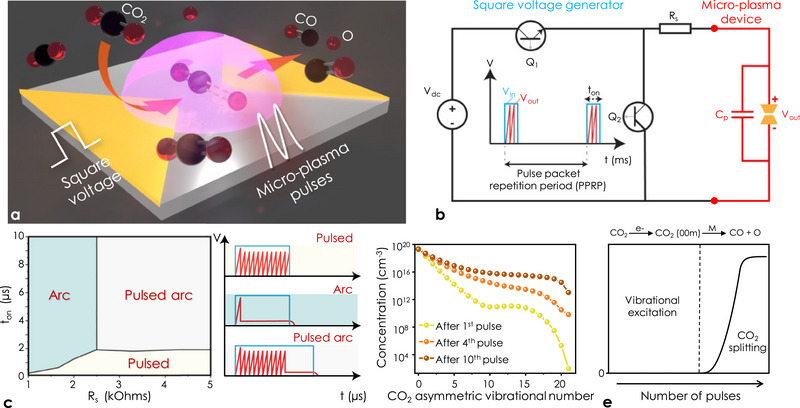
Micro‐plasma device concept, operation modes and principle. a) Schematic of the micro‐plasma device. A square input voltage produces a packet of micro‐plasma pulses to dissociate CO_2_ molecules. b) Square voltage generator circuit. c) Micro‐plasma phase diagram as a function of *t*_on_ and *R*_s_ experimentally obtained for pure CO_2._ Illustrations of the different micro‐plasma phases. d) Effect of the pulse excitation on the CO_2_ asymmetric vibrational distribution functions (model in Experimental Section). As the number of pulses increase, the concentration of higher asymmetric vibrational states rises, leading to CO_2_ dissociation. e) Effect of number of pulses on splitting of CO_2_ into CO.

The micro‐plasma device is composed of two thin‐film electrodes deposited on an insulating substrate, and patterned as two symmetrical v‐shapes, separated by a discharge gap (see **Figure** **1**a and Experimental Section for the description of the device fabrication process flow). The device is driven by a square voltage *V*_in_ applied to one of the electrodes while the second one is grounded. When the amplitude of the square voltage becomes higher than the threshold voltage *V_b_*, which depends on the discharge gap width and feed gas composition, a nanosecond micro‐plasma pulse is discharged across the gap^[^ ^18^ ^]^ where the reaction takes place (Figure 1a). Depending on the driving conditions, several consecutive pulses can be discharged forming a pulse packet.

The square input voltage, defining the pulse packets, is periodically generated by two complementary switching SiC metal‐oxide semiconductor field‐effect transistors (MOSFETs) (Figure 1b), where both the duration of the pulse packet *t*_on_ and the pulse packet repetition period *PPRP* are controlled via a field‐programmable gate array (FPGA) (see Experimental Section). In this circuit configuration, the micro‐plasma device is decoupled from the circuit via a series resistor *R_s_*. The energy per micro‐plasma pulse can be determined from the energy held in the capacitor *C*_p_ (comprising the parasitic capacitance of the plasma device and the voltage probe), which is equal to the ½*C*_p_ *V*_b_^2^ times the number of pulses *N* formed under a single pulse packet. (see Experimental Section for the details regarding the micro‐plasma power and efficiency calculations). In our system, *N* can be increased either by increasing packet duration *t*_on_ at a fixed *R*_s_ or by decreasing *R*_s_ at a fixed *t*_on_. However, *N* cannot be increased indefinitely within a single pulse packet by varying *t*_on_ and *R*_s_ due to the transitions that takes place in the micro‐plasma regimes (Figure 1c). For instance, the minimum *R*_s_ for generating pulsed micro‐plasma was observed to be 1 kOhms. Decreasing *R*_s_ further causes a sufficient portion of the external source current to be injected directly into the plasma to create a sustainable arc that stays fully on after the ignition (Figure 1c). On the other hand, there is also a limit for the maximum attainable duration for *t*_on_, beyond which the micro‐plasma phase transitions to *pulsed arc* (depending on the magnitude of *R*_s_) (Figure 1c). In this phase, both *pulsed* and *arc* phases were sequentially generated within the same voltage pulse packet. This transition infers that the main difference between the pulsed and pulsed arc modes is the residual ion concentration within the discharge volume since the plasma can be sustained at a voltage much smaller compared to *V*_b_ in the pulsed arc phase (Figure 1c). This indicates that after a certain number of discharge events the plasma conductivity increases sufficiently to generate a sustainable low voltage micro‐plasma arc, which can be attributed to the increase of ionic species concentration resulting from the prior pulses, as the pulses form faster compared to the typical ion diffusion times, allowing ionic species to accumulate.

NRP discharges can vibrationally excite CO_2_^[^ ^8^, ^16^, ^19^ ^]^ without thermalizing the plasma, owing to the large mass difference between the electrons and the nuclei,^[^ ^16^ ^]^ allowing electron‐molecule collisions populate the high CO_2_ asymmetric vibrational levels,^[^ ^19^ ^]^ where this process contributes to the generation of vibrationally excited CO_2_ molecules shown by the asymmetric vibrational distribution function (VDFs) given in Figure 1d. This excitation procedure is accompanied by the exchange of vibrational quanta among the excited molecules via ladder climbing mechanism driven by CO_2_(00n) – CO_2_(00n) molecular collisions,^[^ ^11^ ^]^ which eventually leads to splitting of excited CO_2_(00n) into CO and O (Figure 1e). The overall process, known as vibrational excitation mechanism, is the most efficient CO_2_ dissociation pathway since CO_2_ molecules dissociate into CO with an energy cost of ≈5.5 eV, equal to the C = O double bond energy,^[^ ^8^, ^16^ ^]^ unlike the direct‐electron impact dissociation mechanisms that need more than 7 eV to excite CO_2_ into dissociative electronic state.^[^ ^8^ ^]^

During the CO_2_ splitting experiments, one or multiple chips were placed in the flow reactor system given in Figure S1a (Supporting Information) and near pure CO_2_, under atmospheric pressure, was fed into the reactor using a mass‐flow controller (MFC). The DRM measurements were also conducted under atmospheric pressure with a feed gas ratio of CH_4_/CO_2_ = 1, controlled by two separate MFCs dedicated to CO_2_ and CH_4_. The CO and H_2_ production rates were measured by a gas chromatogram (GC) equipped with a thermal conductivity detector (TCD), which also enabled quantification of CO_2_ conversion.

## 3 Results and Discussion

### 3.1 CO_2_ Splitting Performance of All Micro‐Plasma Phases

**Figure 2:**
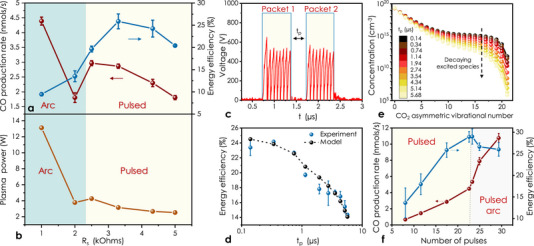
Pure CO_2_ splitting performance of all micro‐plasma phases. a) Effect of resistance R_s_ on the CO production rate and on energy efficiency. b) Plasma power as a function of R_s_. c) Sample of a measured waveform of two pulse packets. d) Effect of inter‐pulse packet time t_p_ on pulsed micro‐plasma driven CO_2_ splitting efficiency. e) Vibrational distribution functions (VDFs) calculated for different inter‐pulse packet times t_p_. f) CO production rate and energy efficiency versus average number of pulses of pulsed and pulsed arc micro‐plasma phases. Error bars were obtained by averaging two to three measurements each conducted on fresh devices.

We investigated the ability of our device to produce CO with three different plasma phases for pulse packet repetition frequency *PPRF* = 1/*PPRP* of 250 Hz and CO_2_ flow rate of 5 mL min^−1^ in order to keep the CO_2_ conversion less than 5% for the kinetic studies. Initially, *pulsed* and *arc* plasma modes were investigated, by arbitrarily fixing the *t*_on_ to 1.26 µs and tuning *R*_s_ between 1–5 kOhms. As shown in **Figure** **2**a, when *R*_s_ was increased from 1 to 2 kOhms, the energy efficiency of the arc micro‐plasma increased meanwhile the CO production rate dropped, which can be attributed to the decreased plasma power (Figure 2b), since the arc micro‐plasma current drops upon increasing *R*_s_, where arc micro‐plasma current (*I*_arc_) is dictated by Ohm's Law (source voltage (*V*_dc_)/*R*_s_). The CO production rate and the energy efficiency increased significantly as the plasma phase transitioned from *arc* to *pulsed* upon increasing *R*_s_ above 2 kOhms, reaching a maximum energy efficiency at *R*_s_ of 3.3 kOhms. For larger *R*_s_, both performance markers dropped despite the plasma power remaining relatively stable (Figure 2b). Since very different CO production rates and energy efficiencies were attained for similar plasma powers, the difference can only be explained by a change in the CO_2_ splitting mechanism. As suggested by control experiments and accurate modelling predictions (based on CO*_2_* vibrational relaxation reaction set developed by Kozak et al.^[^ ^11^ ^]^ and a reduced electric field (E/n) range of 15 ≤ E/n ≤ 50 Td for which the vibrational excitation mechanism is known to be significantly dominant,^[^ ^19^ ^]^ vide infra), the likely explanation for the significant performance increase in *pulsed* plasma regime is the probing of the vibrational excitation mechanism by the repetitive plasma pulses, which gradually and selectively populate higher CO_2_ vibrational energy levels until the molecule disassociates to CO (Figure 1d). To test this hypothesis, two pulse packets with different inter‐pulse packet time *t*_p_ were generated (Figure 2c) to investigate the effect of *t*_p_ on the CO_2_ splitting performance. We observed that the energy efficiency dropped with increasing *t*_p_, demonstrating that the main driving force for the CO_2_ splitting kinetics is the vibrational excitation mechanism that is being fed by each successive pulse and pulse packet (Figure 2d). This conclusion is consistent with previous works linking the effect of pulse excitation time on CO_2_ splitting performance to the presence of carbon dioxide asymmetric vibrational states.^[^ ^10^, ^20^, ^21^ ^]^ Moreover, our efficiency measurements followed a similar trend predicted by the 0D‐kinetic model shown with a dashed line given in Figure 2d. (see Experimental Section and Figure S1b, Supporting Information for the detailed description of the kinetic model and a sample simulation output).

The coherence observed between the experiments and the kinetic model makes it possible to rationalize the effect of *t*_p_ on the reaction efficiency by the CO_2_ asymmetric VDFs calculated for different *t*_p_ (Figure 2e). These VDFs show that the concentrations of CO_2_ (00n) in the lowest energy states remains relatively unchanged while that of highly excited species drop by orders of magnitude as *t*_p_ increases, which is the main reason for the drop in the reaction efficiency as *t*_p_ gets larger. This agrees with the Fridman‐Macheret α‐ model^[^ ^16^ ^]^ which indicates that highly excited CO_2_ molecules have a lower CO_2_ splitting activation energy compared to the low lying states. Thus, the energy efficiency drops once *t*_p_ is larger than the decay time of the highly excited levels (≈100–200 ns for the pulsed micro‐plasma, Figure 2d). Accordingly, this observation also explains why 3.3 kOhms is the *R*_s_ optimum since the average inter‐pulse time between individual micro‐plasma pulses formed under the same packet was ≈75 ns for this particular resistor value.

The pulsed micro‐plasma efficiency was further improved by increasing *N* from 17 to 23, by increasing *t*_on_ from 1.2 to 1.8 µs (see Figure S1c, Supporting Information), which indicates that there was still unreacted CO_2_ (00n) available to be converted into CO by additional pulses. Increasing *N* further caused pulsed phase transition to pulsed arc phase (highlighted in gray in Figure 2f), which decreased the efficiency. However, remarkably, as *t*_on_ increased, the CO production rate tripled with only a 14.5% sacrifice in the energy efficiency. Therefore, the CO_2_ production appears to benefit from the successive arc formation, which however significantly increases the plasma power. On the other hand, the initial rapid energy discharge through pulses, separated by a period shorter than the relaxation lifetime, seems to be sufficient to keep the nuclei and electrons from reaching thermal equilibrium and maintaining the plasma nonthermal and having a relatively high energy efficiency until contribution of the pulsed plasma phase starts to contribute to less than 40% of the total plasma power (Figure S2, Supporting Information). We suggest that sequentially combining *pulsed* and *arc plasma* could be a novel strategy to enhance CO_2_ splitting with negligible losses in the energy efficiency.

### 3.2 Micro‐Plasma Device Scalability and Application to Other Reactions

**Figure 3:**
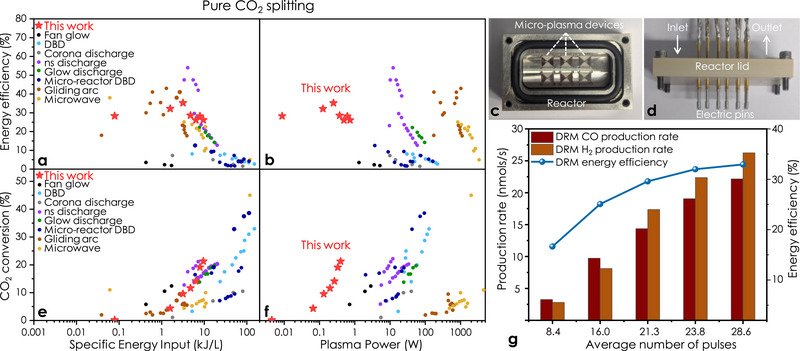
Micro‐plasma device scalability and performance. a) Atmospheric pressure pure CO_2_ splitting energy efficiency benchmark of the current work as a function of specific energy input. b) Atmospheric pressure pure CO_2_ splitting CO_2_ conversion benchmark of the current work as a function of plasma power c) Six decentralized micro‐plasma chips and the micro‐plasma reactor. d) Electrical pins grafted to the reactor lid. e) Atmospheric pressure pure CO_2_ splitting CO_2_ conversion benchmark of the current work as a function of specific energy input. f) Atmospheric pressure pure CO_2_ splitting CO_2_ conversion benchmark of the current work as a function of plasma power. g) Atmospheric pressure DRM performance of a single micro‐plasma device at *PPRF* = 1 kHz, 2.5 mL min^−1^ feed gas flow and feed gas composition CO_2_/CH_4_ = 1.

**Figure** **3**a,b show the effect of SEI and plasma power on the energy efficiency, where the efficiencies were measured for multiple devices (up to 6) that were simultaneously run inside the scaled up micro‐plasma reactor setup given in Figure 3c,d. All chips were driven by generating 7 pulse packets with *t*_on_ = 1.2 µs per chip at *PPRF* = 750 Hz and under a constant pure CO_2_ flow of 2.5 mL min^−1^ to increase the carbon dioxide conversion of the pulsed micro‐plasma phase, which in turn allowed SEI values to be increased by increasing the plasma power. Figure 3a shows that the energy efficiency is maintained at ≈29% for a SEI window ranging from ≈0.1 to 10 kJ L^−1^. Comparable SEI values to other types of plasma systems were achieved with our micro‐plasma device, but with almost ten times lower plasma power below 1 W (Figure 3b), where increasing the SEI also leads to a significant decrease in the energy efficiency for other systems. The low power required to form micro plasmas is a key factor that makes this an alternative lower carbon footprint technology compared to the literature.^[^ ^9^, ^12^, ^13^, ^14^, ^15^, ^22^, ^23^, ^24^, ^25^, ^26^, ^27^, ^28^, ^29^, ^30^, ^31^, ^32^, ^33^ ^]^

The application of pulsed micro‐plasma devices can be extended to other gas phase applications in addition to CO_2_ splitting. We explored DRM, where CO_2_ and CH_4_ were converted into syngas with an efficiency of 33% along with CO/H_2_ ratios close to the theoretical value of 1 (which is suitable for the synthesis of oxygenated chemicals) (Figure 3g). This initial proof of concept demonstrates that the micro‐plasma can activate C‐H bonds at high efficiencies, showing that this low‐power approach can be extended to many other chemistries.

## 4 Conclusion

We introduced a new device that enables controllable generation of different types of low power micro‐plasmas on a small chip. The results on pure CO_2_ splitting showed an energy efficiency of 29% could be achieved for a single device at sub 5% conversion in the pulsed micro‐plasma mode, which is maintained when multiple chips simultaneously driven under optimized conditions to achieve a conversion of 21%, where these results were achieved for plasma powers below 1 W that is at least 10 times lower than other plasma systems with comparable SEI and efficiencies. In addition, it was shown that sequentially combining pulsed and arc micro‐plasma modes leads to 3‐fold increase in the CO production rate compared to pulsed micro‐plasma mode with only a 14.5% sacrifice in the energy efficiency, highlighting that pulsed arc mode can be an alternative approach for improving CO_2_ conversion with negligible losses in the pure CO_2_ splitting energy efficiency. In addition, the applicability of this device to other reactions was demonstrated by running DRM, reaching energy efficiencies up to 33%. This shows the compatibility of this device concept for other reactions while maintaining the low power consumption. The simple fabrication process, high efficiency, versatility, and scalability of our proposed concept could form the basis of a decentralized and electrified chemical production platform that uses CO_2_ as a renewable carbon feedstock and can be fully powered by renewable energy. A future challenge lies in further increasing micro‐plasma throughput while maintaining low power consumption and high energy efficiency. This throughput can likely be improved by optimization of device architecture and reactor design. In addition, future work will also focus on showcasing applicability of these devices to other reactions including methane coupling that is currently under investigation.

## 5 Experimental Section

### Device Fabrication Process Flow

The device fabrication started with cleaning a standard 4‐inch sapphire wafer (Nanjing Co‐Energy Optical Crystal Co. Ltd., 525 µm thickness, 99.995% Purity) with isopropyl alcohol (IPA) and subsequently sputtered a 1.5 µm‐thick tungsten thin film on the cleaned wafer surface (Alliance‐Concept DP 650). The metallic thin film was coated by a 2.5 µm‐thick photoresist (AZ 10XT‐20) and the chip geometry was patterned on the photoresist via photolithography (MLA 150). The photoresist was developed in AZ 400K developer and the wafer was dry etched (Veeco Nexus IBE350). As the final step, the photoresist was removed with REMNP1165 to complete the fabrication (Figure S3, Supporting Information).

### Square Voltage Generation

The square input voltage was generated from a fixed amplitude of 900 V (*V*_dc_), provided by an input DC power supply. A half bridge composed of 2 SiC MOSFETs complementarily controlled generated a square voltage that drives the micro‐plasma device. The control signals defining *PPRF*, *t*_on_, and *t*_p_ of square waves were generated by an Artix‐7 FPGA on the Basys3 development board and supplied to the square voltage generator circuit. The 100 MHz clock of the FPGA provides a control resolution down to 10 ns meanwhile onboard toggle switches allows real‐time tuning of the parameters, *PPRF*, *t*_on_ and *t*_p_, making the system operation very flexible.

### Micro‐Plasma Power and Energy Efficiency Calculations

The energy of each of micro‐plasma pulse was calculated from the discharge of the energy stored in *C*_p_ ($\frac{1}{2}C_{p}V_{b}^{2}$), where *V*_b_ was determined from the average of at least 10 measured waveforms. In the case of arc formation, the plasma stays on after the initial discharge event, resulting in a dissipated power of *I*_arc_ *V*_arc_ during a time *t*_arc_. Therefore, the arc micro‐plasma power *P*_arc_ was calculated by taking both the initial discharge energy of a single pulse and the energy dissipated during the arc period:

(1) $$\begin{array}{c} P_{arc}\;\left(W\right)=PPFR.\;\left(\frac{1}{2}C_{p}V_{b}^{2}+I_{arc}V_{arc}t_{arc}\right)\\ =PPFR.\left(\frac{1}{2}C_{p}V_{b}^{2}+\frac{V_{dc}}{R_{s}}V_{arc}t_{arc}\right)\; \end{array}$$

where *I*_arc_ and *V*_arc_ correspond to the DC the plasma current and the voltage respectively. For the pulsed plasma, the plasma power was calculated based on the total pulse number *N*, which gives the total energy dissipated under a single pulse packet. By multiplying by the pulse repetition rate, the total dissipated power can be calculated:

(2) $$P_{pulsed}\;\left(W\right)=PPFR.\frac{C_{p}}{2}\sum_{i=1}^{N>1}V_{b,i}^{2}\;$$

Lastly, the pulsed arc micro‐plasma power (Figure 2d) was calculated by combining the equations (1) and (2):

(3) $$P_{pulsed\;arc}\left(W\right)=PPFR.\frac{C_{p}}{2}\sum_{i=1}^{N>1}V_{b,i}^{2}+PPFR.\frac{V_{dc}}{R_{s}}V_{arc}t_{arc}\;$$

The energy efficiency (η) was determined by taking the ratio of the chemical power with respect to the power of the micro‐plasma phase:

(4) $$\eta =\frac{r_{total}.\Delta H_{r}}{P_{arc}\;or\;P_{pulsed}\;or\;P_{pulsed\;arc}}\;$$

where *r_total_* is the production rate in µmol/s and *ΔH_r_* is the reaction enthalpy corresponding to either CO_2_ splitting or DRM in J/mol. For pure CO_2_ splitting *r_total_* = *r_CO_* and for DRM *r_total_* = (0.5*r_CO_* + 0.5*r_H2_*).

*Micro‐plasma power and energy efficiency calculations*: The CO production rate was calculated based on the measured CO GC area (*TCDA_CO_*) using the equation (5), where CO_2_ splitting CO production rates were obtained by averaging two to three *TCDA_CO_* measurements each conducted on fresh devices:

(5) $$r_{CO}\;\left(\frac{\mu mol}{s}\right)=TCDA_{\;CO}\;.\;TCRF_{CO}.F$$

where *TCDRF_CO_* is the CO TCD response factor normalized by the GC loop volume and has units of µmol.TCDA_CO_^−1^.ml^−1^ and *F* is the gas flowrate in ml/s. Similarly, the hydrogen production rate was calculated based on (*TCDA_H2_*):

(6) $$r_{H2}\;\left(\frac{\mu mol}{s}\right)=TCDA_{\;H2}\;.\;TCRF_{H2}.F$$

where *TCDRF_H2_* is the H_2_ TCD response factor normalized by the GC loop volume and has units of µmol.TCDA_H2_^−1^.ml^−1^ and *F* is the gas flowrate in ml/s. CO_2_ conversion was calculated as 100%CO yield, i.e., assuming a CO selectivity of 1 (which was supported by the measured O_2_/CO ≈0.5 during initial kinetic studies). The %CO yield was obtained from a dedicated calibration curve for high conversion. Diluting the CO_2_ with inert gases like N_2_, Ar or He both disrupted the stability of the micro‐plasma and increased the complexity of the reaction; thus we ran the reaction with pure CO_2_. However, measuring concentrations in near pure CO_2_ caused the carbon dioxide TCD signal to saturate and become unreliable for conversion calculations, making the estimation of the conversion from the CO yield a more reliable option.

### 0D‐Kinetic Model

The kinetic model shown in this work is a 0D model which calculates the time evolution of number densities of various species as a function of time within a particular volume by using spatially homogenous parameters.^[^ ^11^ ^]^ Mathematically this model is represented as follows:

(7) $$\frac{dn_{i}}{dt}=\;\sum_{j}\left[(a_{j,i}^{R}\;-\;a_{j,i}^{L}\right)]k_{j\;}\prod_{l}n_{l}^{L}$$

where n_i_ is the number density of molecules (cm^−3^) of species i, a^L^_j,i_ is the left hand‐side stoichiometric coefficient of species i in reaction j, a^R^_j,i_ is the right hand‐side stoichiometric coefficient of species i in reaction j, k_j_ is the rate constant of reaction j and n_l_^L^ is the number density of l^th^ species of reaction j. The electron‐impact excitation rate constants *k_e_* were calculated by using the equation below^[^ ^31^ ^]^:

(8) $$k_{e}\left(m^{3}/s\right)=\sqrt{\frac{C}{m_{e}}}\;\int_{0}^{\infty }\varepsilon \;O\left(\varepsilon \right)\;f\left(\varepsilon \right)d\varepsilon$$

where C is the elementary electron charge in Coulombs, m_e_ is the electron mass in kg, ε is the electron energy in eV, O(ε) is the reaction cross section in m^2^ and f(**ε**) is the electron energy distribution function (EEDF) in eV^3/2^. The effects of changing gas composition on the electron‐impact excitation rate constants were neglected since the conversion was always lower than 1% during the kinetic studies. Consequently, EEDF was calculated with BOLSIG+ software^[^ ^34^ ^]^ for pure CO_2_ by using the Phelps database^[^ ^35^, ^36^ ^]^ reaction cross section values compiled in the LXCat website.^[^ ^37^ ^]^ Similar to Kozak,^[^ ^11^ ^]^ O(ε) values of high asymmetric vibrational modes were theoretically estimated by the Fridman approximation^[^ ^16^ ^]^ for the calculation of electron impact excitation of CO_2_ to CO_2_ (00n) for n>1:

(9) $$\sigma_{nm}\left(\varepsilon \right)=\exp\left(\frac{-\alpha \left(m-n-1\right)}{1+\beta n}\right)\sigma_{01}\left(\varepsilon +E_{01}-E_{nm}\right)$$

where *σ_01_* is the reaction cross section for CO_2_ to CO_2_ (001) transition to first asymmetric vibrational level, *E_01_* corresponds to the threshold energy of the transition mentioned before in eV and *E_nm_ = E_m –_ E_n_* is the energy required to transition to asymmetric vibrational level m from level n for m>n in eV. For our calculations we used α = 0.5 as suggested by Fridman^[^ ^16^ ^]^ and β was set to zero as suggested by Kozak.^[^ ^11^ ^]^ The calculated *k_e_* values for electron‐impact excitation reactions were also used for the electron‐impact de‐excitation reactions, as there isn't a clear consensus on the statistical weights of these processes required for the calculation of inverse *k_e_* via principle of detailed balance. The rest of the reaction consisted of the entire CO_2_ vibrational quanta transfer reaction set proposed by Kozak et al.^[^ ^11^ ^]^ along with some neutral‐neutral reactions included in the same work. The vibrational quanta transfer reaction set included the following species given in **Table** **1**:

**Table 1:** Chemical species included in the 0D‐kinetic model.

Species included in the model	Special notation
CO_2_	‐
CO_2_ (010)	V_a_
CO_2_ (020) + (100)	V_b_
CO_2_ (030) + (110)	V_c_
CO_2_ (040) + (120) + (200)	V_d_
CO_2_ (00n, 0 < n < 22)	‐
CO	‐
O	‐
O_2_	‐

In the current work, V_a_, V_b_, V_c_ and V_d_ were assumed to at thermal equilibrium with the CO_2_ (00n) asymmetric vibrational level. Therefore, species such as CO_2_ (01n) that contained quanta at a mode different than the asymmetric stretch were treated as CO_2_ (00n) similar to Kozak. All of the rate constants for vibrational quanta transfer reactions were directly taken from Kozak et al. meanwhile the neutral‐neutral collision rate constants were calculated according to the Fridman‐Macheret α‐model^[^ ^16^ ^]^ in order to account for effects of asymmetric vibrational energy stored in the CO_2_ molecule:

(10) $$k_{n}=A.\exp\left(-\frac{E_{a}-\alpha E_{v}}{T}\right)$$

where *E*_a_ is the activation energy, *E*_v_ is the energy of the vibrationally excited molecule in eV. Similar to calculation cross sections for high asymmetrically excited molecules, the α values were set based on Fridman.^[^ ^16^ ^]^ The micro‐plasma radius *R* was theoretically calculated from the steady‐state diffusion by assuming that the nanosecond micro‐plasma discharge reaches a quasi‐equilibrium shortly after the ionization:

(11) $$-D\nabla^{2}\;n_{e}=G-L=G_{effective}$$

where *n_e_* is the electron density in m^−3^, *D* is the diffusivity of CO_2_ in m^2^/s, *G* and *L* are the electron sources and losses in m^−3^/s that adds up to volumetric effective ionization rate *G*_effective_. The diffusion equation can be simplified by assuming spherical symmetry and a constant G, due to negligible electron recombination during plasma expansion. With these assumptions the equation (11) becomes:

(12) $$\frac{d^{2}n_{e}}{dr^{2}}+\frac{1}{r}\frac{dn_{e}}{dr}+\frac{G_{effect}}{D}=0$$

The equation (12) was solved for the species CO, O, O_2_, CO_2_, CO_2_ (00n), V_a,_ V_b_, V_c_ and V_d_ for T_gas_ = 300 K, *V_b_* = 500 V, P = 1 atm, 6 µm electrode gap (boundary condition n_e_ = 0 at r = *R*):

(13) $$n_{e}=\frac{G_{effect}}{4D}\;\left(1-\frac{r^{2}}{R^{2}}\right)$$

The plasma density profile given in equation (13) predicts that *R* ≈175 µm. Nevertheless, the kinetic model underestimated the efficiency for *R* ≈175 µm even though the model captured the experimentally measured efficiency trend given in Figure 2d. Estimating the semispherical micro‐plasma volume shown in Figure 1 for *R* = 220 µm resulted in a more accurate prediction of the data, where this difference in *R* can be attributed to the v‐shape electrode geometry that wasn't considered in the calculation of the plasma radius. In this work, the equation (7) was numerically solved for the reaction set given in Table 2 via a custom ordinary differential equation (ODE) solver coded in MATLAB. Since the vibrational relaxation rate constants are calculated for 300K in Kozak's work,^[^ ^11^ ^]^ the gas temperature was also set as 300K for the sake of consistency. The experimental results (Figure 2d) were modelled for more than one possible reduced electric field (E/N) and *n_e_* combinations in an attempt to address the uncertainties arising from the complex nature of the micro‐plasma. Consequently, we estimated that 15 ≤ E/N ≤ 50 and peak *n_e_* is between 1 × 10^16^ cm^−3^ – 2.5 × 10^16^ cm^−3^ for a micro‐plasma pulse with a volume of *V*_p_ = 2.5×10^−5^ cm^3^. The theoretical energy efficiency values used to plot the dashed‐line given in Figure 2d were calculated as the following:

(14) $$n_{theoretical}=\frac{PPRF.\left[CO\right].V_{p}}{N_{A}\;}\;\frac{\Delta H_{CO2}}{P_{pulsed}}$$

where [CO] the CO number density in cm^−3^ calculated by the model for each specific *t*_p_ and *N*_a_ is the Avogadro's number.

**Table 2:** Reaction set.

Code	Reaction	Refs.
X1	e^−^ + CO_2_ (00n) ←→ e^−^ + CO_2_ (00 m) (0 ≤ n ≤ 20, 1 ≤ m ≤ 21, m > n)	[20,40,41References 40,41 has been cited but is not present in the reference list. Please provide details or delete the citation from main text.]
X2	e^−^ + V_a_ ←→ e^−^ + CO_2_ (00 m) e^−^ + V_b_ ←→ e^−^ + CO_2_ (00 m) e^−^ + V_c_ ←→ e^−^ + CO_2_ (00 m) e^−^ + V_d_ ←→ e^−^ + CO_2_ (00 m) (1 ≤ m ≤ 21)	[20,40,41]
V1	CO_2_ (00 m) + CO_2_ (00n) ←→ CO_2_ (00m‐1) + CO_2_ (00n+1) (0 ≤ n ≤ 20, 1 ≤ m ≤ 21, m > n)	[20]
V2	V_a_ + CO_2_ (00 m) → CO_2_ (001) + CO_2_ (00m‐1) V_b_ + CO_2_ (00 m) → CO_2_ (001) + CO_2_ (00m‐1) V_c_ + CO_2_ (00 m) → CO_2_ (001) + CO_2_ (00m‐1) V_d_ + CO_2_ (00 m) → CO_2_ (001) + CO_2_ (00m‐1) (1 ≤ m ≤ 21)	[20]
V3	CO_2_ + CO_2_ (00 m) → V_a_ + CO_2_ (00m‐1) CO_2_ + CO_2_ (00 m) → V_b_ + CO_2_ (00m‐1) CO_2_ + CO_2_ (00 m) → V_c_ + CO_2_ (00m‐1) CO_2_ + CO_2_ (00 m) → V_d_ + CO_2_ (00m‐1) (1 ≤ m ≤ 21)	[20]
VT1	CO_2_ (001) + M → V_a_ + M CO_2_ (001) + M → V_b_ + M CO_2_ (001) + M → V_a_ + M CO_2_ (001) + M → V_b_ + M CO_2_ (001) + M → V_c_ + M CO_2_ (00 m) + M → CO_2_ (00m‐1) + M (2 ≤ m ≤ 21)	
VT2	V_a_ + M → CO_2_ + M V_b_ + M → CO_2_ + M V_c_ + M → CO_2_ + M V_d_ + M → CO_2_ + M	[20]
N1	CO_2_ + M → CO + O + M	[20]
N2	O + O → O_2_	[20]
N3	CO_2_ + O → CO + O_2_	[20]
N4	CO + O + M → CO_2_ + M	[20]

## Conflict of Interest

The authors declare no conflict of interest.

## Supporting information



Supporting Information

## Data Availability

The data that support the findings of this study are openly available in Low‐Power Tunable Micro‐Plasma Device for Efficient and Scalable CO2 Valorization Raw data at [DOI], reference number 15194671.
