# Supplementary material for: Low‐Power Tunable Micro‐Plasma Device for Efficient and Scalable CO2 Valorization
Source: Adv Sci (Weinh). 2025 Jun 30;12(35):e07687. doi: 10.1002/advs.202507687 (PMC12463112; doi:10.1002/advs.202507687)
Supplement: Supplementary file 1 — Supporting Information [file ADVS-12-e07687-s001.docx]

Supporting Information

Low-Power Tunable Micro-Plasma Device for Efficient and Scalable CO_2_ Valorization

Bartu Karakurt, Hongkeng Zhu, Onder Soydal, Guangyu Sun, Jeremy S. Luterbacher* and Elison Matioli*

Bartu Karakurt, Hongkeng Zhu, Onder Soydal, Guangyu Sun and Elison Matioli

Institute of Electrical and Micro-engineering, Ecole Polytechnique Fédérale de Lausanne (EPFL), Lausanne, Switzerland
E-mail: [elison.matioli@epfl.ch](mailto:elison.matioli@epfl.ch)

Bartu Karakurt, Jeremy S. Luterbacher
Institute of Chemical Sciences and Engineering, Ecole Polytechnique Fédérale de Lausanne (EPFL), Lausanne, Switzerland

E-mail: [jeremy.luterbacher@epfl.ch](mailto:jeremy.luterbacher@epfl.ch)

**Supplementary Figures**

**Figure S1.** a) The flow reactor setup contains two Brooks SLA580 low flow rate (0-10 ml/min) mass-flow controller (MFC) that allows sending pure CO_2_/CH_4_ into the micro-plasma reactor at a constant flow rate. CO production rate and CO_2_ conversion was measured by the Perkin Elmer AutoSystem XL GC meanwhile the areas under the each signal were integrated by the dedicated GC software. During the measurements, square wave characteristics (*PPRF*, *t_on_*, and *t_p_*) were controlled by the Basys3 FPGA unit meanwhile waveforms were recorded via TektronixTHDP0200 voltage probe connected to the Tektronix MDO3104 oscilloscope b) Average number of micro-plasma pulses corresponding to various *t_on_* values (*V*_in_ = 900 V, *PPRF* = 250 Hz, *R*_s_ = 3.3 kOhms) were measured under 5 ml/min atmospheric pressure CO_2_ flow. As can be seen, average number of pulses saturates around 6 µs and increasing *t_on_* further than this point causes pulsed phase to transition to pulsed arc phase c) A sample simulation output.

**Figure S2.** The individual contributions of arc and pulsed micro-plasma to the pulsed arc phase were controlled by changing the *t*_on_ parameter for a fixed *R*_s_ of 3300 Ohms. Even though both the number of pulses and the arc duration were simultaneously increased, increasing *t*_on_ always augmented the arc character of the pulsed arc phase. However, the increase in the arc character didn’t significantly decrease the efficiency of the CO_2_ micro-plasma phase until reaching *t*_on_ = 8 µs, where, at this point, the energy injected by the arc phase severely took off as a result of *ca.* 5 folds increase in the *V*_arc_. Despite the origins of the increase observed in *V*_arc_ is not very clear, the increased of the arc character of the pulsed arc phase significantly decreased the CO_2_ splitting efficiency (down to 15%) when the contribution of the pulses to the overall energy was pushed down to less than 40%.

**Figure S3.** Micro-plasma chip fabrication process flow.
